# Observation of Blood Pressure as an Aid to Diagnosis

**Published:** 1916-12

**Authors:** Charles U. Patterson

**Affiliations:** Houston, Texas


### Observation of Blood Pressure as an Aid to Diagnosis.

BY DR. CHARLES U. PATTERSON, HOUSTON, TEXAS.

The diagnosis of disease has been and always will be the most difficult problem the honest, conscientious physician has to solve. It is therefore to be expected that he will ever be on the alert to discover some new means to this end, and in his eagerness to obtain it will grasp and hold for a time many things that lead him into error.

When he has learned that there is no royal road to diagnosis and is able to analyze the various signs and symptoms and corrolate them, then and then only can he make a rational use of the various aids to diagnosis. If there was one single symptom, physical or laboratory finding that was always present in a certain disease and absent when that disease did not exist, diagnosis would be easy.

Our literature is full of "signs," laboratory findings, etc., that have been considered pathognomonic of a disease until thousands of costly errors have proven them false. The failure to recognize malaria, because the plasmodium did not appear in the blood, has cost many a life. The positive assurance that the child did not have measles because there were no Koplik spots has put many a doctor to shame. Tenderness over McBurney's point even with a high white count has wrecked the professional aspirations of many an over-enthusiastic surgeon, and caused many persons the loss of a perfectly good appendix. None of these or other fads, that have been followed blindly, can do more harm when improperly applied than the present fad of taking blood pressure.

I am aware that it is somewhat unwise and unorthodox to speak of taking blood pressure as a fad, but I am quite sure it will not be so common in ten years as it is now, but it will be of far greater value when done.

In speaking of taking of the blood pressure by means of a sphygmomanometer as a fad, I do not wish it to be understood that I do not consider this an instrument of value. There can be no question of its value when properly used; in fact, I consider its use nearly as important as that of the clinical thermometer, but the estimation of the blood pressure does not any more diagnose the case than the taking of the temperature.

A blood pressure may be high enough to endanger life without regard to its cause, just as a fever may be a menace to life.

The over-enthusiasm for pressure observations at this time is largely due to commercialism. These instruments are easily and cheaply made and therefore sell at an enormous profit, making it possible for the factory to send out fairly well-informed men to demonstrate them and impress their importance upon the doctors. In this manner blood pressure has become an entity instead of a symptom of any of a number of diseases, or a condition that may exist in the absence of any disease.

The insurance companies have added no little to the rapidly increasing demand for these instruments by insisting on a reading of the blood pressure of their applicants. I am not at all sure that the taking of the afternoon temperature would not be of more value than the blood pressure, and there can be no doubt that temperature will be correctly taken and blood pressure will not be correctly observed in but very few cases.

The sphygmomanometer is not an instrument of scientific precision, but an instrument of estimation, and is only valuable in the hands of men who can use this estimate at its true value. Like the thermometer, it may warn us that there is something wrong and lead us to an investigation which may result in finding the cause of the disturbance. A rising blood pressure is usually compensatory. This is nature's plan of conserving certainl bodily functions, and the lowering of it by drugs might be attended with disastrous results. While this is true, it sometimes happens that the process is carried to a dangerous height and must be reduced just as a fever that has reached a dangerous elevation.

We have long ago learned the folly of trying to reduce a fever that was only a normal reaction to certain diseases, and we have now learned that blood pressure is just as essential to certain conditions as fever and is even more dangerous to tamper with. A gradual rise in pressure as one advances in years is much safer than a lowering or even stationary one.

The systolic pressure may be taken with a fair degree of accuracy if the following conditions are observed: The patient must be at rest and entirely free from mental strain or embarrassment, and the muscles thoroughly relaxed. It is very difficult to judge of the patient's state of mind, but the muscular tension can be approximated by touch. Then there is the nervous patient who stiffens the muscles just when the band is almost tight and sends the reading up ten or twenty or even more. In these cases the osculatory method is not so good as the palpatory, for with the finger on the pulse we can detect a sudden stiffening of the muscle and either not read until it is normal or allow for the tension. Rest, posture and the placing of the band has been well described in all the text-books on this subject.

Diastolic pressure is more difficult to estimate, and when estimated its significance is often a perplexing question. The normals that exist in health cannot obtain in disease. There is very often a resistance to the arterial pressure that is overcome before it reaches the other side, and there is no need for an increased venous pressure. The various methods of estimating the diastolic pressure have been so well described that it is not necessary for me to discuss them any more than to say that none of them are exact and one is about as good as the other. The most important factor in the estimate is the judgment of the reader. Nearly all operators recognize four distinct sounds as the pressure is released from somewhere above the systolic pressure to the complete silence that follows the reading of the diastolic pressure. The third and fourth of these sounds blend so closely together that it is not always possible for me to tell when the third has ended and the fourth begun nor can I tell just when the fourth is going to end. The fact is that it continues while the reading on the dial or column cover several mm. Hg., and might be read at one place as well as the other.

The oscillatory method has as many more objections. The whole question of diastolic reading is nicely summed up by Mackenzie on page 138 of his third edition, where he says, "As the cause of these oscillations is not understood, the attempts to estimate the mean or diastolic pressure from them is little better than guesswork, and in practice it will more likely tend to mislead than to give reliable information." The pressure in the arm may differ widely from that of the leg. Hill and Rowland have found as much as 100 mm. Hg., and not dependent on sclerosis.

While the range of blood pressure is wide and all sorts of mistakes are made in the readings and their significance, it still has a place as a useful aid if carefully used and not accepted at more than its real value. When we feel the pulse and discover what we think is an increased tension, it is well to use this instrument to verify our opinion. If the sphygmomanometer shows a reading above the normal for the age, we should first determine whether it is above normal for that particular individual, and if so, look for and remove the cause. The first thing to consider in the absence of any history bearing on this is toxemia from overeating, overindulgence in tobacco or other narcotics, or constipation.

If the patient has been a big eater, an immoderate smoker and an intemperate drinker, he can be greatly benefited by a proper diet and the reduction of his tobacco and liquor. If he has constipation, that should be corrected as far as possible, not by the constant use of purgatives, but by the smallest dose of a laxative that will keep the bowels open when on a coarse diet. After excluding the above causes of high pressure, I think renal congestion or sclerosis is next the most frequent cause of high pressure. Renal congestion is frequently produced from the causes given above, but it may come on independently. A temporary congestion may raise the pressure temporarily, and it will lower again when the kidney is restored to its normal condition, thus making frequent readings necessary to determine whether this is functional and temporary or organic and permanent. In either case the increased pressure is compensatory. It is nature's plan to continue the kidney function, which could not be done under normal pressure, and the duty of the physician is to assist in this by giving the patient a diet that will lessen the work of the kidney and see that the skin and bowels are doing their share of the work.

In the conditions above mentioned, there is no reason why the diastolic pressure should be greatly increased and no effort should be made to restore the usual normals by either trying to lower the systolic or raise the diastolic by drugs given for that purpose alone. In these conditions the two pressures do not require any definite ratio to each other in order to perform their proper function. Hypertension due to plumbism can usually be determined by the usual symptoms, but one must not expect to always have a severe colic, drop wrist, etc., but the blue line and stippling blood are nearly always present, and this together with the occupation and place of living will complete the diagnosis. In chronic cases of lead poisoning with hypertension albumen is sometimes found, and if we are not careful we may mistake it for nephritis. The high pressure so often found in syphilis can be determined by the usual tests and history.

It is often quite difficult to differentiate the conditions already given from general arteriosclerosis, and in fact impossible except by exclusion as above indicated. The estimation of blood pressure in arteriosclerosis is attended with many difficulties. An arteriosclerosis may be local or general. Certain vessels or groups of vessels may be hardened while others are normal. An apoplexy may occur soon after a low reading or a high reading may exist for years without any apparent injury. It has been generally conceded that a long continued high pressure will end in nephritis or the so-called vascular nephritis, which is supposed to distinguish this type from that in which the kidney is primarily at fault and the increased pressure the result. I shall not attempt to split hairs as to these divisions, but will say that I am of the opinion that more of the kidney cases are due to the toxic forms of high pressure than to arteriosclerosis, and that the sphygmomanometer has its greatest use in the detection of these toxic cases. I have already spoken of the difficulty encountered in attempting to estimate the pressure in general sclerosis, besides the muscular tension we have the added resistance of a hardened artery. It should be remembered that we are not trying to measure the elasticity of the artery, but the force of the blood stream as it passes through the artery.

The condition of the artery represents another unknown quantity. We have looked on this condition of the arteries as one that comes on normally as persons grow older, and this is in a measure true so far as the reading of the pressure gauge is concerned, but if the vessels are hardened sufficiently to cause an increased force to drive the blood through them there will be an increased demand made on the heart to accomplish this work, and it must gradually hypertrophy to meet these demands. It therefore frequently happens that the calcareous degeneration of the artery progresses until its elasticity is destroyed and its movements no longer assist in the circulation, and whatever force the blood stream has is derived from the heart and largely the left ventricle. How can we measure the blood stream that is thrown from a heaving ventricle and comes into the radial artery as a sledge-hammer pulse? If the arm is raised above the head the fingers and even the whole hand will soon show evidences of deficient arterial blood supply. Evidently a high pressure should not be so easily overcome by gravity.

The estimation of the force and stability of the heart by the blood pressure is attended with so many difficulties that few, if any, can hope to arrive at a correct conclusion. In a very pronounced myocardial insufficiency the pressure might be low enough to cause a suspicion of heart lesion, but usually the ordinary clinical symptoms would be so inevident that a reading of blood pressure would not add to what was already known. A pulse may be irregular in time and tension without indicating disease, but in disease we find a variety of arythmetic conditions that cannot be measured as to tension. Where there is one hard beat followed by one, two or more weaker beats and each of these of different tension it has, so far as I know, been the custom to measure the blood pressure by the hardest beat, as it is the last cut off by the armlet. In this way the pressure is estimated for one-half, onethird, or one-fourth of the time but does not give any mean pressure, and a diastolic pressure estimated on such a reading certainly is the merest guesswork.

The attempt to arrive at any idea of the condition of the heart by taking a blood pressure at rest and then again after exercise is worse than useless and would certainly lead anyone attempting it into serious error. No possible standard could be fixed for such a reading and the variations must depend on the irritability of the heart and the patient's habits as to exercise.

A hard-working man or an athlete may run a block or climb several flights of stairs with very little change in his pressure, even though he has a badly diseased heart, while the neurasthenic woman with a good heart may show a wide difference in the readings.

